# Cultural Context and Mental Health: A Kenyan Elite Athlete’s Perspective

**DOI:** 10.1007/s40279-025-02283-6

**Published:** 2025-09-02

**Authors:** Andrew Kirkland, Anna C. Whittaker, Michael Boit, Stephen Chinn, Michael Crawley, David Ndetei, Paul Ochieng, Ewan Stirling, Irene Chesire

**Affiliations:** 1 Faculty of Health Sciences and Sport, University of Stirling, Union Street, Stirling, FK9 4LA Scotland, UK; 2 Kenyatta University, Nairobi, Kenya; 3 Department of Anthropology, Durham, UK; 4 University of Nairobi and Africa Institute of Mental and Brain Health (AFRIMEB), Nairobi, Kenya; 5 Strathmore University, Nairobi, Kenya; 6 Department of Behavioural Sciences, Moi University School of Medicine, Eldoret, Kenya

## Abstract

The causes of mental ill health in elite athletes are complex, influenced by socioeconomic and cultural factors. These factors are important in shaping discussions surrounding the mental health of athletes and the design of subsequent interventions to support them. However, such consideration is rare, particularly when considering the mental health of athletes in low- and middle-income countries. Therefore, this Current Opinion draws on behavioural change science and multi-disciplinary expertise in elite sport, medicine, health, psychology, coaching and anthropology in the context of elite runners in Kenya. The material conditions in this country are reflected in the prevalence of poor mental health and treatment availability. We explore the mental health of elite Kenyan runners within this context and provide recommendations surrounding the treatment of mental health conditions in elite sport globally. We conclude that a consensus on the mental health of elite athletes must be informed by contextual factors, including affordability, appropriateness, availability, and accessibility of mental health services relating to local conditions. This context-specific approach could be expanded to mental health in settings other than sport such as youth organisations.

## Key Points

**Table:** 

We highlight the importance of recognising context and cross-cultural differences when exploring mental health in elite sport.
Reductionist and clinical approaches to mental health interventions are unlikely to be impactful, particularly in low- and middle-income countries.
A consensus on the mental health of elite athletes should be informed by contextual factors, including the affordability, appropriateness, and availability and accessibility of mental health services relating to local conditions.

## Is it Possible to Reach a Consensus on the Mental Health of Elite Athletes?

The (IOC) Consensus Statement on Mental Health in Elite Athletes [1] reflected an important step forward in the understanding and management of mental health in sport. However, it is important to recognise that mental health and strategies for management were presented through an implicit but contested biomedical discourse [2–6] with the presumption that there is a consensus on the methods to diagnose mental health pathologies and that resources are available to implement management strategies. Whilst it was recognised that there is a paucity of evidence surrounding cross-cultural differences in the meanings and manifestations of mental health symptoms and disorders, we suggest that a consensus must reflect “the diversity and complexity of mental health needs of individuals or populations” [7, p. 1533].

The purpose of presenting our opinion is to encourage reframing of the prevalent mental health discourse represented in the IOC Consensus Statement. Specifically, there needs to be a greater focus on and more research in low-resource environments, particularly when the capacity to treat mental health conditions is usually limited regardless of a country’s socioeconomic status [7]. East Africa is a relevant region to explore the mental health of athletes because it represents a low-resource region that has been successful in achieving international success, predominantly in endurance running. Whilst very little research has been conducted in this area, our expert opinion reflects the convergence of perspectives from coaching, psychiatry, medicine, anthropology, sport and lived experiences in East Africa.

Specifically, we believe that any consensus must account for cross-cultural perspectives and the capacity to deliver effective interventions in low-resource environments. We explore the Kenyan context, believing that many of the lessons learnt from there are translatable to other low-resource environments globally.

## Introduction to Cross-Cultural Differences in Sport

The International Olympic Committee (IOC) consensus statement on mental health in elite athletes recognised that cross-cultural differences in meanings and manifestations of mental health symptoms and disorders were under-addressed [1]. We wish to expand upon this point by suggesting that a biopsychosocial understanding of the causes of mental health issues is essential to create sustainable and effective solutions to meet mental health challenges in sport and other settings. We illustrate these points by reference to sociocultural factors relating to Kenyan runners.

Cross-cultural differences, including life values, motivations and epistemological variations, are fundamental to how people think about mental health. Emotional expression and idioms of distress [8, 9] are conditioned by culture, and therefore will differ across various groups of athletes. Contextual factors should be central to debates surrounding mental health in sport and must be at the forefront of how we discuss and tackle mental health globally. Biological reductionism and a clinical focus can help in a small number of cases. However, we suggest that the IOC Consensus Statement and the wider mental discourse in sport should be framed more comprehensively. This framing needs to reflect the complex and context-specific nature of mental health, particularly in low- and middle-income countries.

## Mental Health of Kenyan Runners

Kenya is a country of extreme inequality, in which healthcare provision for most of the population is limited. Within this context, runners, especially from the Nandi, Keiyo and other Kalenjin subtribes from North Rift Counties, have been global leaders in endurance running since the 1960s. The reasons for such success are complex, with biological, psychological, socioeconomic and environmental factors interacting to produce consistently high numbers of world leading athletes from a small geographical area [10]. A range of factors have been explored in attempts to understand the success of Kenyan athletes, including genetic predisposition, maximal oxygen uptake, metabolic efficiency, haematological profile, diet, living at altitude, walking to school and motivations linked to economic advancement [10]. Additionally, research suggests that elite Kenyan athletes possess distinct ethnic and environmental backgrounds compared with the general Kenyan population [11]. Rather than attributing success to any single factor, a complex causal nexus [2] exists. The interplay of factors across multiple disciplinary domains provides a more integrative explanation for Kenyan running excellence, transforming their lives and those of their communities through the income running success brings [12].

Importantly, developing runners believe they can follow in the footsteps of previous Kenyan champions. They see the significant material wealth that success can bring to communities where income, food security and employment are precarious. Consistent with the intensity of motivation theory [13], large numbers of Kenyan runners are sufficiently optimistic about success to justify the investment of considerable energy into running. This theory suggests that the level of effort an individual invests in a task is influenced by both their perceived importance of the goal and their expectancy of success. Higher motivation intensity arises when a goal is viewed as valuable and achievable. Because elite runners are concentrated and prominent within a relatively small geographic region, aspiring athletes from similar backgrounds view success as attainable. Running is rarely a leisure pursuit; rather the motivational orientation of the Kenyan elite runners is on task orientation and a fear of failure [14].

Similar to the Ethiopian context [15], the association between running and mental health is ambivalent. Running can be an important way for young people to maintain a hopeful and speculative outlook on the future but can require withdrawal from society and feelings of futility at other times. However, there is growing concern about the mental health of Kenyan runners. Whilst prevalence rates of diagnosable mental health conditions amongst them are unknown, anecdotal evidence of alcohol misuse, anxiety, depression and suicide are relatively common. These occur in communities where the overall prevalence of mental illness is high at approximately 45% [16, 17]. Further, within the Kenyan community, the prevalence of anxiety, alcohol misuse, depression and suicide attempts have been reported to be 15.7%, 11.7%, 12.6% and 16.4%, respectively [16]. Cause is consistent with multidimensional, and intersectional models of mental health [7], and intertwined with cultural idioms of distress [8, 9]. Idioms of distress are culturally distinct expressions and experiences of emotional suffering that may not align with conventional Western medical classification systems. These idioms are likely to be related to ontological perspectives of what mental health is to rural Kenyans, in whom religiosity and cultural beliefs are likely to affect the appraisal of feelings of mental malaise.

Whilst under-explored empirically, challenges relating to education attainment, poor financial management, cultural transitions, exploitation and doping may interact to affect the mental health of runners. Our experiences in Kenya suggest life as an athlete is precarious. A lack of self-management, dips in form, illness and injury are ever-present factors that mean the chances of success are low. In addition, we suggest that social comparison and status are important to athletes from these communities. Champion runners are viewed as role models within their communities. This may lead to status anxiety, or social defeat [18], where there are psychological and physiological consequences of a perceived loss of social status. Runners may be unable to deal with the stressors related to the demands of international competition, or with the increased social standing that success brings. Poor mental health, in addition to affecting socio-emotional abilities, can also limit runners’ potential to support sustainable development goals in the North Rift Counties. For example, income generated from running success is commonly used by athletes to support a quality education, provide local infrastructure, and develop partnerships with local government.

Whilst the IOC Consensus statement [1] recognises the biopsychosocial nature of mental health, it is still dominated by biological reductionism and clinical pathological focus. Such orientation may be less relevant to low- and middle-income countries in which resources to support mental health are negligible and referral pathways to expert clinicians are almost non-existent. Research in the communities where Kenyan elite athletes originate from suggests that only 3.6% of people with mental disorders receive treatment [11], typically only for those most seriously ill. For many East African runners, there is little conceptualisation of talent or natural ability. Rather, hard work, improvement of socioeconomic status, spiritual state and moral fortitude are considered more explanatory of success. In respective studies [11, 19], it was found that socioeconomic factors are a major driving force behind elite athletes’ exceptional performances. Social suffering and deeper malaise are often self-explained as weakness, the result of divine intervention or conveniently ignored until it is too late. Thus, poor awareness, high stigma and a low perceived need for the treatment of poor mental health are present [20]. Clinical interventions, primarily relating to the treatment of symptoms rather than the cause, are unlikely to be accessed through expensive private provision. Clinical treatments can be important in reducing individual suffering but have limited effects at a population level [7].

## Lessons to be Learnt on a Global Scale

When poor mental health consistently occurs in specific sports contexts, the cause is rarely situated within the individual but in the social system [7]. Changing that means understanding the determinants of current and desired behaviours of relevant actors and the biopsychosocial context in which their behaviours occur [21]. In other words, to be effective, treatments need to be evidence guided and have the potential to address the cause [2]. There is inconsistency in how referral processes are defined, resourced and accessed between sports programmes, even in developed nations [22]. Many mental health issues are sub-clinical or unrecognised [23, 24]. Barriers to change are considerable. Where toxic sporting environments exist, programmes are often led by directors and coaches who are unlikely to accept that they need to change [25, 26]. Conflicting interests in federations and governing bodies mean that systemic issues are often left unaddressed [27]. Suggesting interventions to improve mental health such as raising awareness, education or updating best practice can be of value. However, well-resourced systems of care, capable of responding to athletes’ needs, are required if they are to be helpful [28]. This requires motivation and funding, usually from central bodies with budgetary constraints and a lack of expertise. Preaching to the converted is often the result of many interventions.

While there are no easy answers, most reductionist and clinical approaches to intervention are unlikely to be impactful. Rather, research models such as the Theoretical Domain Framework [21] are advocated to better understand the complex biopsychosocial nature of mental health. This integrative model synthesises multiple psychological and behavioural theories to systematically assess and understand contextual factors influencing behaviour. There is sufficient evidence to demonstrate the framework can be used to develop healthcare interventions [29], including in low-to-middle income countries [30], if it is used as a flexible rather than rigid framework [31]. The Theoretical Domain Framework was built recognizing that to be able to change collective and individual behaviours, it is important to understand what the drivers of these behaviours are [32]. By doing so, sustainable solutions to tackle mental health challenges in sport can be identified. Such solutions will not be found when only the voices of clinical experts are considered. Rather, a diverse variety of stakeholders, including non-specialist providers [33] such as coaches, must be central to debates surrounding consensus statements in mental health. This is particularly important in communities where awareness of and professional skills in the provision of mental health services are lacking. We suggest the use of the three-component COM-B system [32] to address this. This has the potential to identify cross-cultural factors that interact in complex ways to drive health behaviours (B) and influence mental health. These components are:

- *Capability*: which refers to the knowledge, skills and physical abilities of individuals.
- *Motivation*: relates to goal-orientated behaviours, but also emotion regulation and conscious decision making.
- *Opportunity*: relates to external social factors that make the behaviour possible or prompt it.

By developing a better understanding of these behavioural drivers, a more culturally sensitive evidence base surrounding mental health can be developed. Doing so would help identify context-specific barriers to and facilitators of behavioural change. The ‘opportunity’ component of COM-B typically is sociological, a disciplinary perspective that often exists outside the scope of a pathogenic lens, but it is important one when exploring mental health. The voices of people in low- and middle-income countries and particularly the very communities which athletes stem from must also be heard if Olympic values relating to global social development through sport are to occur.

## Conclusions

To conclude, reaching a consensus on how to support the mental health of elite athletes is problematic, particularly when such a consensus is based on a contested Western biomedical model of what mental health is. Furthermore, many of the treatments suggested in the IOC Consensus Statement [1] are only available to athletes who have access to well-resourced healthcare systems and sporting systems that can respond adequately to their needs [28]. Rather, identifying dominant factors that influence mental health and the most appropriate “points of attack for modification and adjustment” [34] in relation to the resources available is advised. To summarise, we recommend greater consideration of the following factors when examining mental health in sport:

1. Co-creation of the evidence base, in which the perspectives of stakeholders outside clinical domains are given more prominence.
2. A greater focus on establishing treatments and interventions that reflect the complex, context-specific and culturally sensitive nature of mental health.
3. Deeper awareness of and focus on transdiagnostic processes and treatments [7] that can be delivered in sustainable ways.
4. Development of treatments that can be realistically accessed by and tailored to the needs of athletes and that can be delivered by non-specialists.

This cultural contextual approach could be expanded to understanding and treating mental health in global settings other than sport, such as in education or youth organisations.
